# Development of Silicone Elastomer-Based Composite Films Containing Ibuprofen and Functional Additives

**DOI:** 10.3390/ijms27167446

**Published:** 2026-08-20

**Authors:** Mari Atabekyan, Zoya Farmazyan, Nelly Avagyan, Vigen Topuzyan, Stepan Grigoryan, Gohar Khachatryan, Karen Khachatryan

**Affiliations:** 1 The Scientific Technological Centre of Organic and Pharmaceutical Chemistry NAS RA, 26, Azatutyan Str., Yerevan 0014, Armenia; atmari@yandex.ru (M.A.); zoefa2000@yahoo.com (Z.F.); nelli.avagyan80@gmail.com (N.A.); vtop@web.am (V.T.); grigstepan@yahoo.com (S.G.); 2 Department of Food Analysis and Quality Assessment, Faculty of Food Technology, University of Agriculture in Krakow, Al. Mickiewicza 21, 31-120 Krakow, Poland; gohar.khachatryan@urk.edu.pl; 3 Laboratory of Nanotechnology and Nanomaterials, Faculty of Food Technology, University of Agriculture in Krakow, Al. Mickiewicza 21, 31-120 Krakow, Poland

**Keywords:** ibuprofen, polysiloxane composites, alkoxysilane cross-linkers, apparent drug release, synthetic skin membrane, preliminary membrane permeation, copper oxide nanoparticles, sea buckthorn oil

## Abstract

Silicone elastomers are attractive matrices for transdermal drug delivery systems, but the controlled release of poorly water-soluble drugs from hydrophobic silicone networks remains challenging. Medical-grade silicone elastomers are generally regarded as chemically stable, biologically inert, and highly biocompatible polymer matrices, which supports their use in biomedical and pharmaceutical materials. Here, ibuprofen-loaded silicone/polyol composite films were prepared from hydroxyl-terminated polydimethylsiloxane (PDMS-OH) using glycerol- and 1,2-propylene glycol-derived alkoxysilane cross-linkers and amino-terminated PDMS as a metal-free room-temperature-vulcanising catalyst. The effects of cross-linker composition, glycerol, PEG 200 and selected functional additives on film formation, morphology, apparent ibuprofen release and preliminary Strat-M^®^ permeation were evaluated. FTIR analysis indicated no covalent reaction between ibuprofen and the silicone network, but suggested hydrogen-bonding interactions with polyol-rich domains, particularly in glycerol-containing systems. Raman mapping supported ibuprofen incorporation within the films, while SEM showed phase-separated microdomains whose morphology depended on the formulation. Apparent release into 0.9% NaCl at 37 °C was formulation-dependent over 72 h. The optimised F-9 film showed approximately 83% cumulative apparent release, whereas the F-10 film containing copper oxide nanoparticles and sea buckthorn oil showed the highest numerical cumulative apparent release, approximately 94%. Kinetic analysis of the apparent release data supported a mainly diffusion-controlled contribution, modulated by hydrophilic microdomains. These results provide preliminary materials-development evidence that silicone/polyol films can be used to tune apparent ibuprofen release and merit further optimisation for local topical or transdermal applications; however, efficient skin permeation and biological performance require dedicated validation.

## 1. Introduction

The skin, as the largest and one of the most biologically active organs of the human body, performs a wide range of vital functions. It acts as a protective barrier against ultraviolet radiation, chemical agents, and microorganisms, while also playing a crucial role in the regulation of water balance. Owing to its large surface area (1.5–2 m^2^) and rich blood supply, the skin represents an attractive route for systemic therapy and drug absorption [1,2]. However, the outermost layer of the epidermis, *stratum corneum*, constitutes the primary barrier to diffusion and remains the major limitation of transdermal drug delivery systems [3,4]. This challenge is particularly pronounced for hydrophobic drugs with low aqueous solubility, such as ibuprofen [3,5].

Ibuprofen is a widely used nonsteroidal anti-inflammatory drug with proven analgesic and anti-inflammatory efficacy. Nevertheless, its systemic (oral) administration is associated with serious adverse effects, including gastrointestinal bleeding, as well as an increased risk of myocardial infarction and stroke [6,7]. An effective strategy to mitigate these drawbacks is the transdermal delivery of ibuprofen, which enables localised therapeutic action at the site of inflammation while maintaining low plasma drug concentrations. This approach is especially advantageous for the long-term management of chronic pain and inflammatory conditions [8,9].

To enhance drug permeation across the skin, a wide range of penetration enhancers have been investigated in transdermal systems, including glycols, fatty acids, surfactants and natural oils [10,11]. In the present work, glycerol, 1,2-propylene glycol and PEG 200 were used primarily to modify the hydrophilicity and microstructure of the silicone matrix. In addition, naturally derived materials such as Aloe vera gel (*Aloe barbadensis Miller*) and sea buckthorn oil (*Hippophae rhamnoides*) were considered as functional additives because of their reported skin-conditioning, moisturising and anti-inflammatory-related properties [12,13,14,15]. However, their biological activity was not directly assessed in this study; therefore, their role is discussed as formulation functionality requiring further dedicated biological validation rather than as a confirmed therapeutic effect.

In addition to natural additives, inorganic nanoparticles, particularly copper oxide (CuO) nanoparticles, have been explored in polymeric and biomedical materials because copper-containing species may provide antimicrobial effects and can participate in biological processes relevant to tissue repair. In the present work, these properties are treated as a rationale for material design rather than as direct evidence of biological activity of the final films [16].

Polydimethylsiloxane (PDMS) is widely employed as a polymeric base for transdermal films due to its excellent biocompatibility, mechanical flexibility, and high permeability to oxygen and moisture [17]. Medical-grade silicone elastomers are generally regarded as chemically stable, biologically inert and highly biocompatible materials, which have supported their extensive use in biomedical devices, pharmaceutical formulations and drug delivery systems [18]. Cross-linked silicone films are also extensively used in clinical practice for skin regeneration and scar management, where they function as occlusive systems that hydrate the *stratum corneum* and modulate collagen synthesis [19]. These properties make PDMS a highly attractive platform for the development of topical and transdermal drug delivery material platforms.

This study presents a silicone/polyol platform intended to advance silicone films from passive protective coverings toward active ibuprofen-loaded transdermal prototypes. The present manuscript focuses on synthesis, morphology, drug distribution, apparent release, and preliminary permeation; biological claims related to antimicrobial, anti-inflammatory, or regenerative activity are therefore discussed as potential functionalities requiring further validation. No cytotoxicity, antimicrobial, wound-healing, or other biological assays were performed at this stage.

Compared with previously reported PDMS-based ibuprofen systems, the distinguishing feature of the present approach is not the use of PDMS alone, but the combination of polyol-derived alkoxysilane cross-linkers within situ retained glycerol/1,2-propylene glycol and PEG 200 domains. The specific advance demonstrated in this study is the correlation between cross-linker chemistry, polyol-rich microdomains, spectroscopic drug distribution and apparent release behaviour in a room-temperature-cured silicone/polyol film platform.

In this formulation strategy, amino-terminated PDMS acts as a metal-free curing component; therefore, no organotin or platinum catalyst was used during film preparation. Ibuprofen was used primarily as a model hydrophobic drug to probe loading, spectroscopic distribution, apparent release and preliminary membrane permeation in the newly developed silicone/polyol network, rather than to claim optimisation of a finished ibuprofen patch.

## 2. Results and Discussion

### Curing Characteristics and In Vitro Apparent Drug-Release Studies

**Figure 1:**
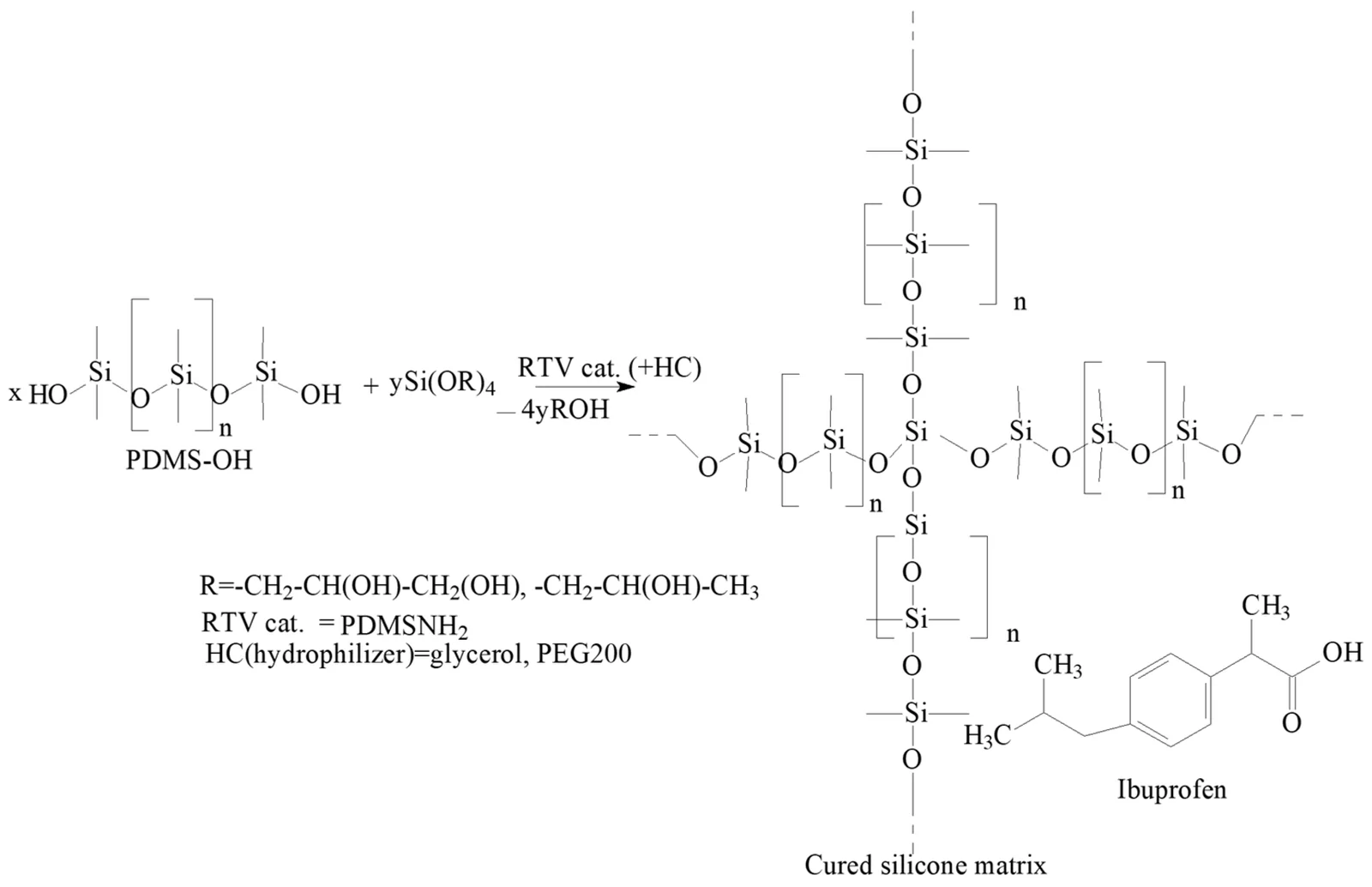
Scheme of the silicone films synthesis.

The compositions of the ibuprofen-loaded silicone composite films are presented in Table 1. The influence of the chemical structures of both ibuprofen and the cross-linking agents on film curing duration and appearance was evaluated. The well-known polycondensation reaction of TEOS with PDMSOH produces a cross-linked silicone elastomer and generates ethanol, which evaporates from the system. In contrast, when Si-GL-4, Si-PGL-4, or Si-GL:PGL (2:2) are used as cross-linkers, the reaction follows a similar mechanism but releases polyols (glycerol and 1,2-propylene glycol) (Figure 1). These polyols remain within the silicone matrix acting as pore-forming agents and hydrophilic reservoirs, thereby strongly influencing the apparent release behaviour and ultimate properties of the composites.

The F-1 film crosslinked within 24 h, whereas the F-4 and F-7 films crosslinked within 5 min. Adhesion of the films to paper and plastic substrates was also tested. The F-1 film exhibited strong adhesion, while no significant adhesion was observed for the F-4 and F-7 films. All three films demonstrated good stability, with no visible changes upon over three months of observation.

**Figure 2:**
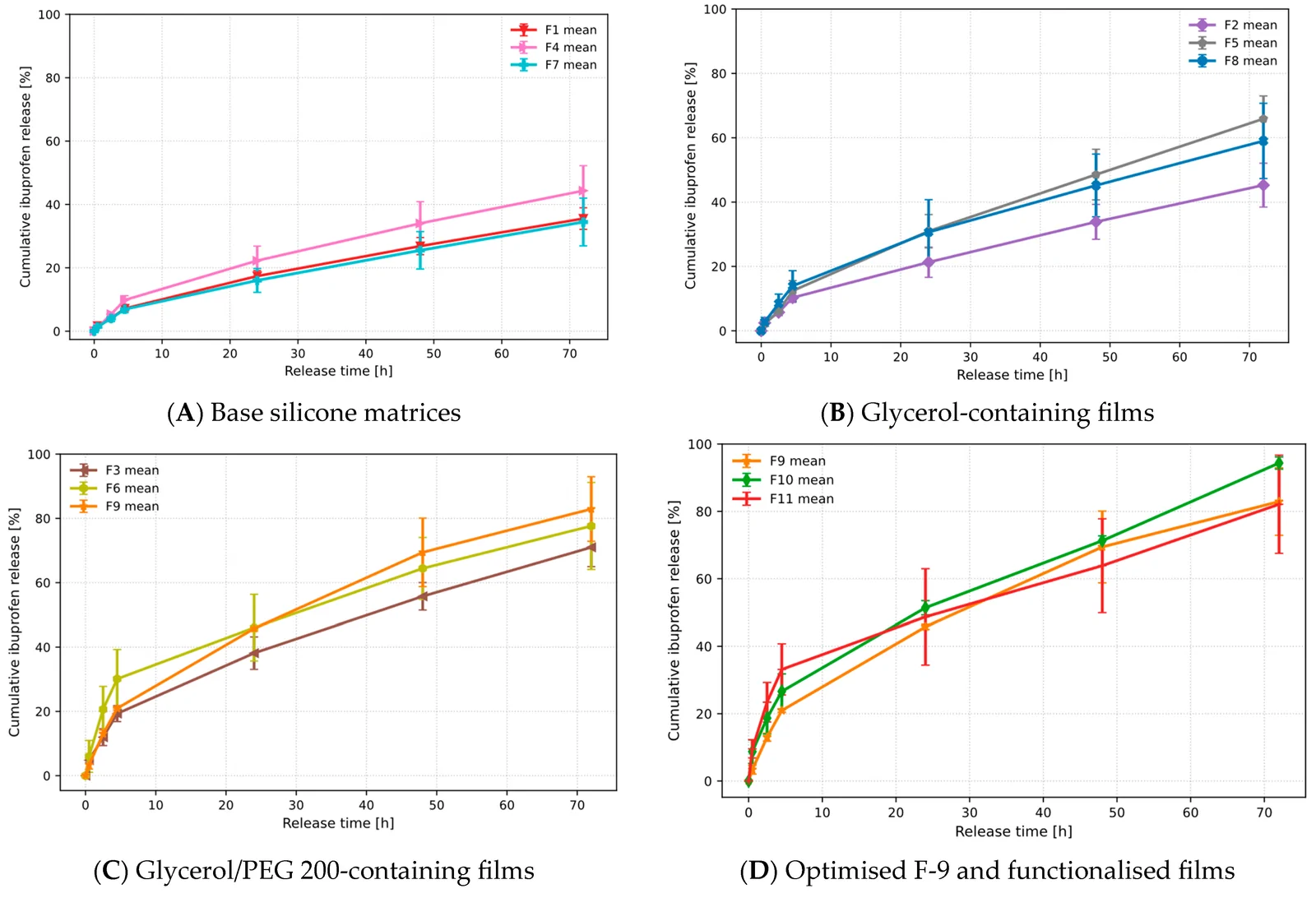
Cumulative apparent ibuprofen release from silicone-based composite films grouped according to formulation function: (**A**) base silicone matrices without additional hydrophilic additives; (**B**) glycerol-containing films; (**C**) glycerol/PEG 200-containing films; and (**D**) optimised F-9 formulation and functionalised F-10/F-11 films. Data are presented as mean ± SD (*n* = 3). F-6 is shown as a fragmented structural-control sample and was not used for direct mechanistic ranking of intact films.

Apparent ibuprofen release into 0.9% NaCl was monitored for 72 h at 37 °C using complete medium replacement at each sampling point (Figure 2). Because the experimentally estimated solubility of ibuprofen in 0.9% NaCl at 37 °C was low (approximately 1.19 mg·100 mL^−1^, i.e., 0.0119 mg·mL^−1^), the profiles are interpreted as apparent release under defined saline-replacement conditions rather than as unequivocally sink-controlled release [20]. The replicate mean ± SD values used for the error bars are provided in Table S1. The final grouped release profiles are presented in Figure 2.

Consequently, the apparent release data are used for relative comparison of formulations tested under the same protocol, and not as absolute intrinsic release rates obtained under confirmed sink conditions.

The apparent release behaviour reflects the balance between the hydrophobic PDMS-rich phase and the hydrophilic polyol-rich domains formed during curing. Silicone is intrinsically hydrophobic; therefore, incorporation or in situ generation of glycerol, 1,2-propylene glycol, and PEG 200 is expected to increase water uptake, create hydrophilic microreservoirs, and facilitate ibuprofen diffusion. However, because ibuprofen has limited aqueous solubility in saline, the cumulative apparent release values should be discussed together with the medium-replacement protocol and not interpreted as absolute sink-controlled dissolution.

For this reason, differences between formulations are discussed as numerical trends in apparent release under identical saline-replacement conditions unless statistical significance is specifically stated.

For the glycerol-containing films F-2, F-5, and F-8, the same nominal PDMSOH:glycerol mass ratio was used. The addition of glycerol increased the apparent release compared with the corresponding non-glycerol matrices, but the effect depended strongly on the cross-linker composition. After 72 h, F-2 showed apparent release of 45.27 ± 6.82%, releasedF-5 showed apparent release of 65.88 ± 7.11%, and F-8 showed apparent release of 59.01 ± 11.69% ibuprofen. This indicates that the polyol phase alone does not determine the release rate; the cross-linker-derived network structure and the distribution/connectivity of hydrophilic domains also play a major role.

To further increase apparent ibuprofen release, part of the glycerol phase was replaced with PEG 200 while maintaining the total polyol content at approximately 44 wt%. The glycerol-to-PEG 200 mass ratio was adjusted to 3:1, resulting in F-3, F-6, and F-9 films. F-3 required 3 days for cross-linking, whereas F-9 cured within 5 min and formed a continuous film. F-6 required 4 days and produced fragmented cross-linked pieces rather than a continuous membrane; therefore, it is reported as a structural-control sample and excluded from direct ranking of intact films. The PEG-containing systems showed higher cumulative apparent release than the corresponding glycerol-only or non-polyol formulations: F-3 showed apparent release of 70.99 ± 6.00%, F-6 showed apparent release of 77.63 ± 13.54%, and F-9 showed apparent release of 82.91 ± 10.04% ibuprofen after 72 h.

**Figure 3:**
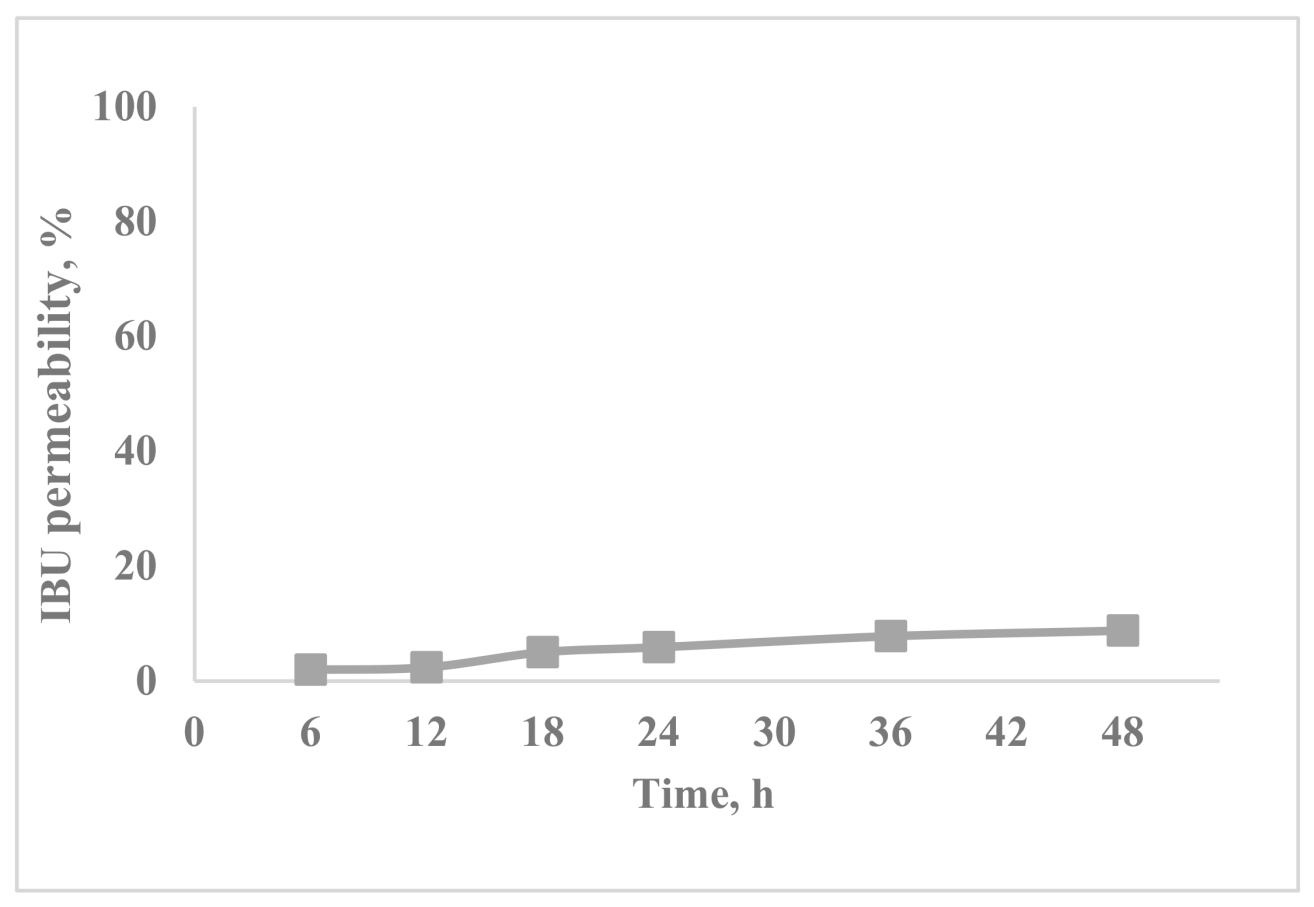
Cumulative permeated amount of ibuprofen from the F-9 composite film using a Franz diffusion cell apparatus with a Strat-M^®^ membrane and 0.9% NaCl solution (mean ± SD, *n* = 3).

To determine whether the increased apparent drug release was associated with composite film swelling, swelling studies were conducted. The swelling degree reached 172% on the first day, gradually decreasing to 85% at 48 h and 13% at 72 h, which may be associated with leaching of water-soluble components and redistribution of water within the silicone/polyol network. The cumulative Strat-M^®^ permeation profile of the F-9 film is presented in Figure 3.

Figure 3 shows preliminary membrane permeation of ibuprofen from the F-9 composite film using a Franz diffusion cell apparatus, a Strat-M^®^ synthetic skin diffusion membrane, and 0.9% NaCl solution at 37 °C.

The cumulative permeated amount of ibuprofen after 48 h was approximately 9%.

This low permeated fraction indicates that the present F-9 film should not yet be regarded as a validated system for systemic transdermal administration. At this stage, the formulation is more appropriately considered a local topical/dermal delivery prototype or a material requiring further optimisation before true transdermal performance can be claimed.

Accordingly, the 48 h Strat-M^®^ result is treated as a baseline permeability indicator of the matrix and not as evidence of a clinically effective transdermal patch.

Compared with currently used topical ibuprofen formulations and previously reported transdermal or nano-delivery approaches, the present silicone/polyol films are not yet directly comparable in terms of clinically validated skin delivery performance. Their potential advantage lies in the material design: an elastomeric silicone/polyol network formed by room-temperature curing and containing hydrophilic domains that can modulate apparent ibuprofen release. At the same time, the low Strat-M^®^ permeation, absence of biological validation, and lack of mechanical and long-term stability data remain important limitations, so the system should currently be considered a material platform for further optimisation rather than a ready topical or transdermal product [5,8,9,17].

Based on the optimised F-9 formulation, which combined rapid polymerisation with high numerical apparent ibuprofen release under the applied saline-replacement protocol, two additional composite films were prepared to explore functional modification of the silicone/polyol platform. The F-10 film was obtained by incorporating a blend of sea buckthorn oil (*Hippophae rhamnoides*, approximately 3 wt%) and copper oxide (CuO) nanoparticles (approximately 1 wt%) into the F-9 base before curing. Sea buckthorn oil and CuO nanoparticles were selected as complementary functional additives; however, their contribution to biological performance should be confirmed in dedicated assays.

F-9 was selected as the base formulation because it provided the most favourable practical compromise among the intact glycerol/PEG 200-containing films: rapid curing, formation of a continuous film, high numerical cumulative apparent release and Raman-confirmed ibuprofen distribution.

This stepwise design allowed the effects of CuO/sea buckthorn oil and Aloe vera to be evaluated against the same base silicone/polyol matrix without changing the underlying cross-linker/polyol composition.

For the F-11 composite film, a polysaccharide-rich Aloe vera extract (*Aloe barbadensis*, ~4% *w*/*w*) was incorporated as the primary additive. Aloe vera was selected because literature reports describe moisturising and skin-conditioning properties, but these effects were not tested for the present film.

The functional additives further modified the apparent release profiles. The optimised F-9 formulation released 82.91 ± 10.04% ibuprofen after 72 h, whereas F-10, containing CuO nanoparticles and sea buckthorn oil, showed the highest numerical cumulative apparent release (94.36 ± 1.79%) under the applied saline-replacement protocol. The smaller SD observed for F-10 at 72 h suggests comparatively reproducible release from this formulation. This numerical increase relative to F-9 may be associated with changes in matrix morphology, increased interfacial area, and the presence of oil- and particle-containing dispersed domains that alter drug partitioning and diffusion pathways. F-11, containing Aloe vera extract, showed apparent release of 82.11 ± 14.58% after 72 h and exhibited a more pronounced early-stage release, as also reflected by the larger Q4.5/Q72 contribution. Because no biological assays were performed, the Aloe vera-containing system is described here as a moisturising/skin-conditioning prototype rather than as a biologically validated regenerative material.

Because cytocompatibility, irritation, antimicrobial and wound-healing tests were not performed, the practical biological significance of CuO nanoparticles, sea buckthorn oil and Aloe vera in the final films remains speculative at this stage.

Overall, the apparent release data support the versatility of the silicone/polyol for tuning apparent ibuprofen release under the applied experimental conditions through cross-linker selection and incorporation of hydrophilic or functional additives. The results also indicate that the apparent release mechanism is governed by diffusion through a heterogeneous elastomeric matrix containing polyol-rich domains, rather than by a single homogeneous dissolution process.

To compare the apparent release profiles quantitatively, the cumulative apparent release data were fitted to zero-order, first-order, Higuchi, and Korsmeyer–Peppas models. The Higuchi model was used as the principal comparative model for the full apparent release profiles, whereas the Korsmeyer–Peppas model was applied to the initial release region (F ≤ 0.6) to estimate the release exponent n [21,22,23,24]. The main kinetic parameters are summarised in Table 2. The corresponding Higuchi fitted profiles are shown in Figure S1, the complete fitting statistics are provided in Table S2, and simple two-phase release descriptors are listed in Table S3. The generally high R^2^ values for the Higuchi model support a dominant diffusion-controlled contribution, whereas the Korsmeyer–Peppas exponents indicate non-ideal/anomalous transport in several formulations, consistent with release from hydrophilic microdomains embedded within the silicone matrix.

Exploratory one-way ANOVA of Q72 values did not indicate statistically significant differences within the predefined formulation groups shown in Figure 2 at *p* < 0.05 (groups A–D: *p* = 0.213, 0.071, 0.421 and 0.331, respectively). The same conclusion was obtained when AUC0–72 values were analysed as supportive whole-profile descriptors (groups A–D: *p* = 0.176, 0.147, 0.197 and 0.706, respectively). Therefore, the apparent-release differences are interpreted as numerical trends rather than as statistically confirmed differences in intrinsic release performance.

**Figure 4:**
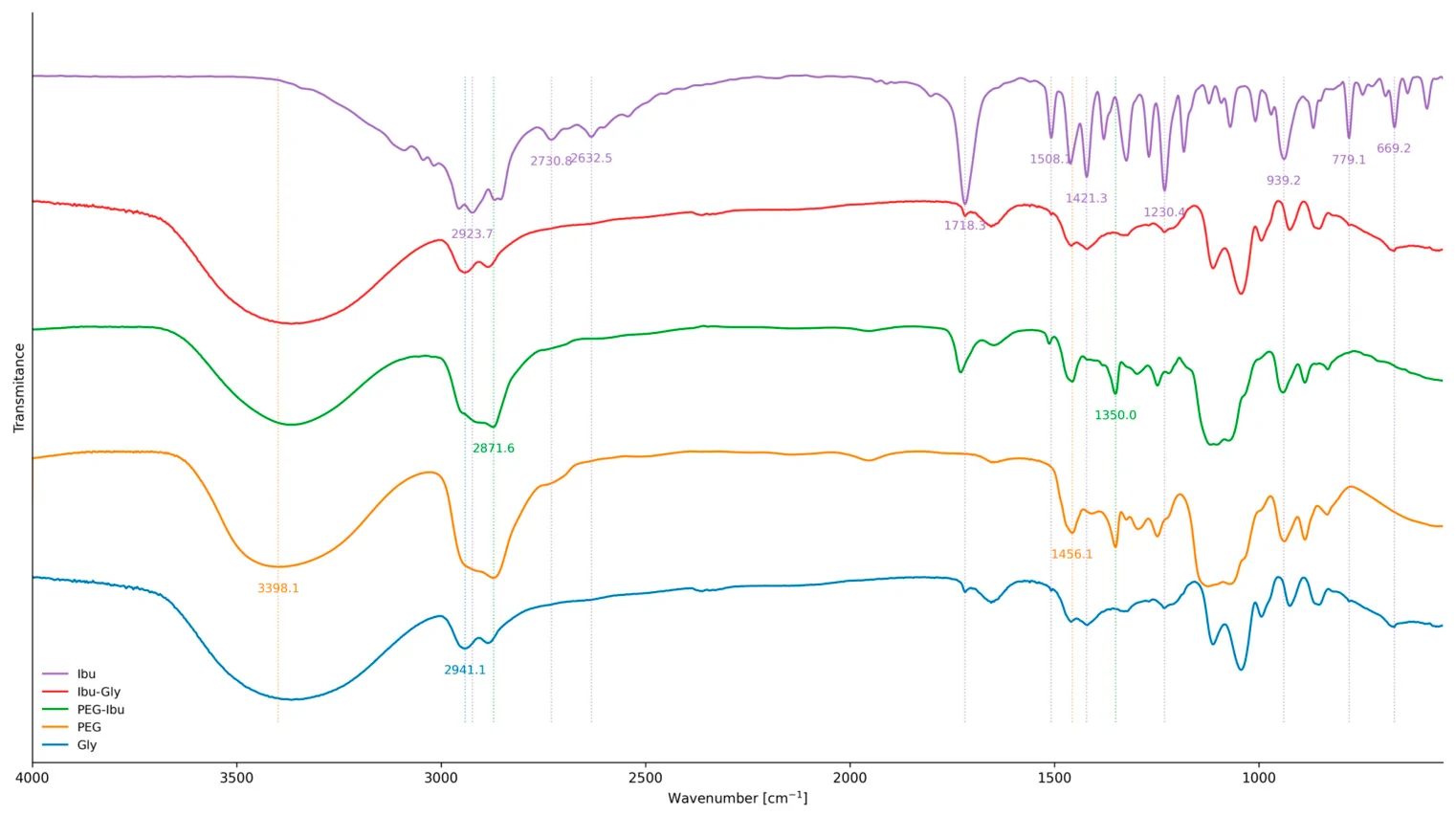
FTIR spectra of IBU, PEG 200, glycerol and IBU + glycols mixtures.

FTIR studies were conducted to understand the differing apparent drug-release profiles from composite films with identical base compositions but different hydrophilic agents, and to probe potential drug-trapping chemical interactions. The characteristic infrared spectrum of ibuprofen is well-documented, featuring a strong carbonyl (C=O) absorption band at approximately 1721 cm^−1^. Key secondary peaks include a broad band at ~2958 cm^−1^, assigned to the asymmetric C–H stretching vibration of methyl (CH_3_) groups, and a peak at ~1230 cm^−1^, attributed to C–C stretching vibrations (Figure 4) [25,26]. To confirm the successful incorporation and probe the molecular state of glycerol within the composite films, FTIR analysis was conducted. The spectra exhibited the characteristic peaks of glycerol, confirming its presence in the polymer matrix. The identified peaks include a broad band at ~3300 cm^−1^ (O–H stretching), along with bands at 2934 cm^−1^ and 2879 cm^−1^, assigned to the asymmetric and symmetric CH_2_ stretching vibrations, respectively. A peak at 1413 cm^−1^ corresponds to CH_2_ bending. Two strong peaks in the region between 1300 and 1000 cm^−1^ are characteristic of C–O stretching vibrations: one at 1108 cm^−1^ (secondary alcohol C–O stretch) and another at 1030 cm^−1^ (primary alcohol C–O stretch). Additional peaks were identified at 993 cm^−1^ (C–C stretching vibration), 851 cm^−1^ (C–C–O symmetric stretching), and 674 cm^−1^ (O–H wagging) [27]. The corresponding FTIR spectra of the reference components and physical mixtures are presented in Figure 4.

The FTIR spectrum of pure polyethylene glycol 200 (PEG 200) showed a broad O-H stretching band at approximately 3397 cm^−1^, consistent with hydrogen-bonded hydroxyl groups. Aliphatic C-H stretching bands were observed at 2934 and 2872 cm^−1^, while the dominant C-O-C ether stretching band appeared at approximately 1124 cm^−1^. Additional CH_2_ deformation, wagging and rocking bands confirmed the expected spectral features of hydrated PEG 200 [28].

The FTIR spectra of pure ibuprofen, pure glycerol, and their physical mixture reveal distinct changes (Figure 4). In the mixture, the intensity of ibuprofen’s main bands—especially the C=O band—decreases significantly, accompanied by band broadening. Notably, the broad O–H band shifts and widens considerably. These changes are consistent with hydrogen-bonding interactions between the hydroxyl groups of glycerol and the carboxyl group of ibuprofen. This hydrogen bonding may contribute to partial retention of ibuprofen molecules within the polymer matrix, slowing their diffusion and apparent release, which may partly account for the lower apparent drug release observed from glycerol-containing films (F-2, F-5, and F-8).

In contrast, the spectrum of the ibuprofen–PEG 200 mixture showed much weaker changes, indicating negligible interaction. Reduced drug–polyol trapping in PEG-containing systems may facilitate drug diffusion, in line with the higher apparent release observed from such films as F-3, F-6, and F-9 (Figure 4).

The presence and distribution of ibuprofen in the composite were studied using Raman spectroscopy. The spectrum shows the main characteristic bands of the drug. The most important marker was the intense peak at ~1608 cm^−1^**,** attributed to the aromatic C=C bond stretching vibration, which is in complete agreement with data reported in the literature for pure Ibuprofen. Another characteristic band was observed at approximately 833 cm^−1^ and assigned to aromatic ring breathing. Additional characteristic bands (~1455, ~1181, ~745 cm^−1^) and their assignments are presented in Table 3 [29,30,31]. Raman spectroscopy was also recorded for each individual component of the composite formulation; the results are summarised in Table 3. The selected Raman assignments used for component identification and ibuprofen mapping are listed in Table 3.

**Figure 5:**
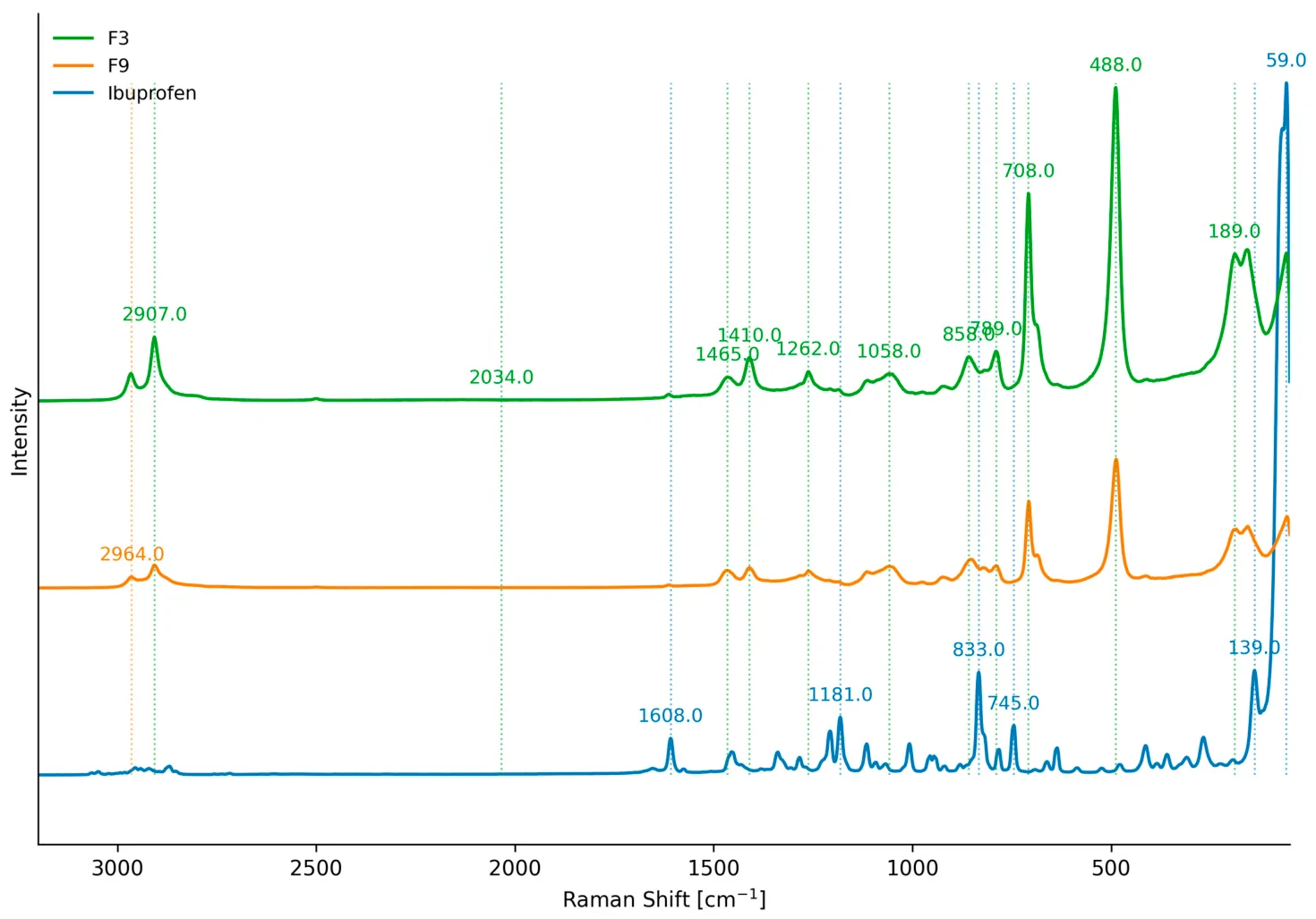
Raman spectra of ibuprofen-containing composite films and reference components used for marker-band selection.

For Raman mapping of the synthesised films, the four synthesised films F-3, F-9, F-10, F-11, and the ibuprofen band at approximately 1608 cm^−1^, assigned to aromatic C=C stretching, were selected as the principal marker. This band was chosen because it was characteristic of ibuprofen and showed suitable separation from the main bands of the other formulation components. The presence of the principal ibuprofen bands in the spectra of F-3 and F-9 confirmed drug incorporation and enabled spatial assessment of its distribution within the films (Figure 5).

**Figure 6:**
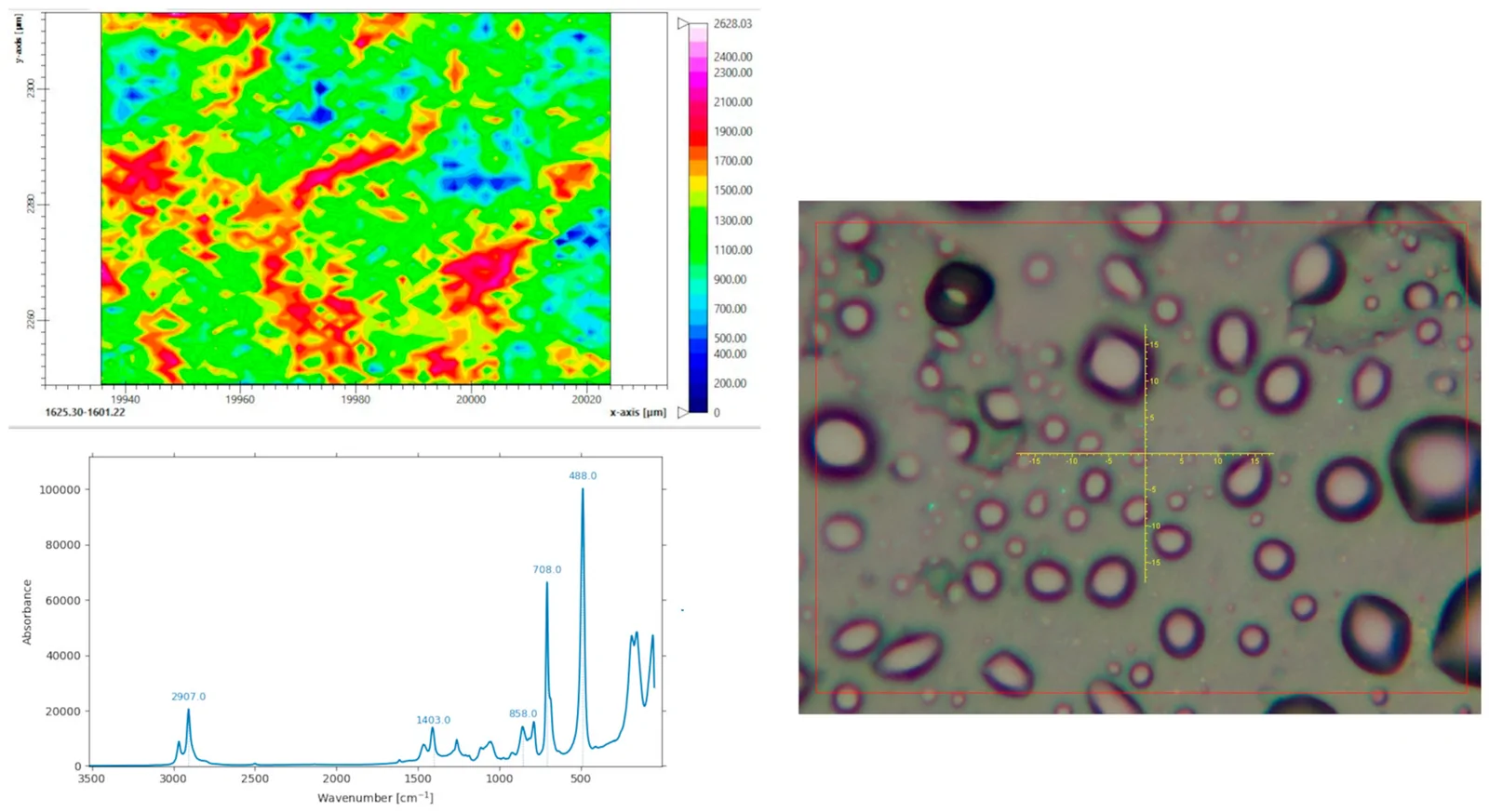
Raman spectrum, chemical map and optical image of the F-3 film.

**Figure 7:**
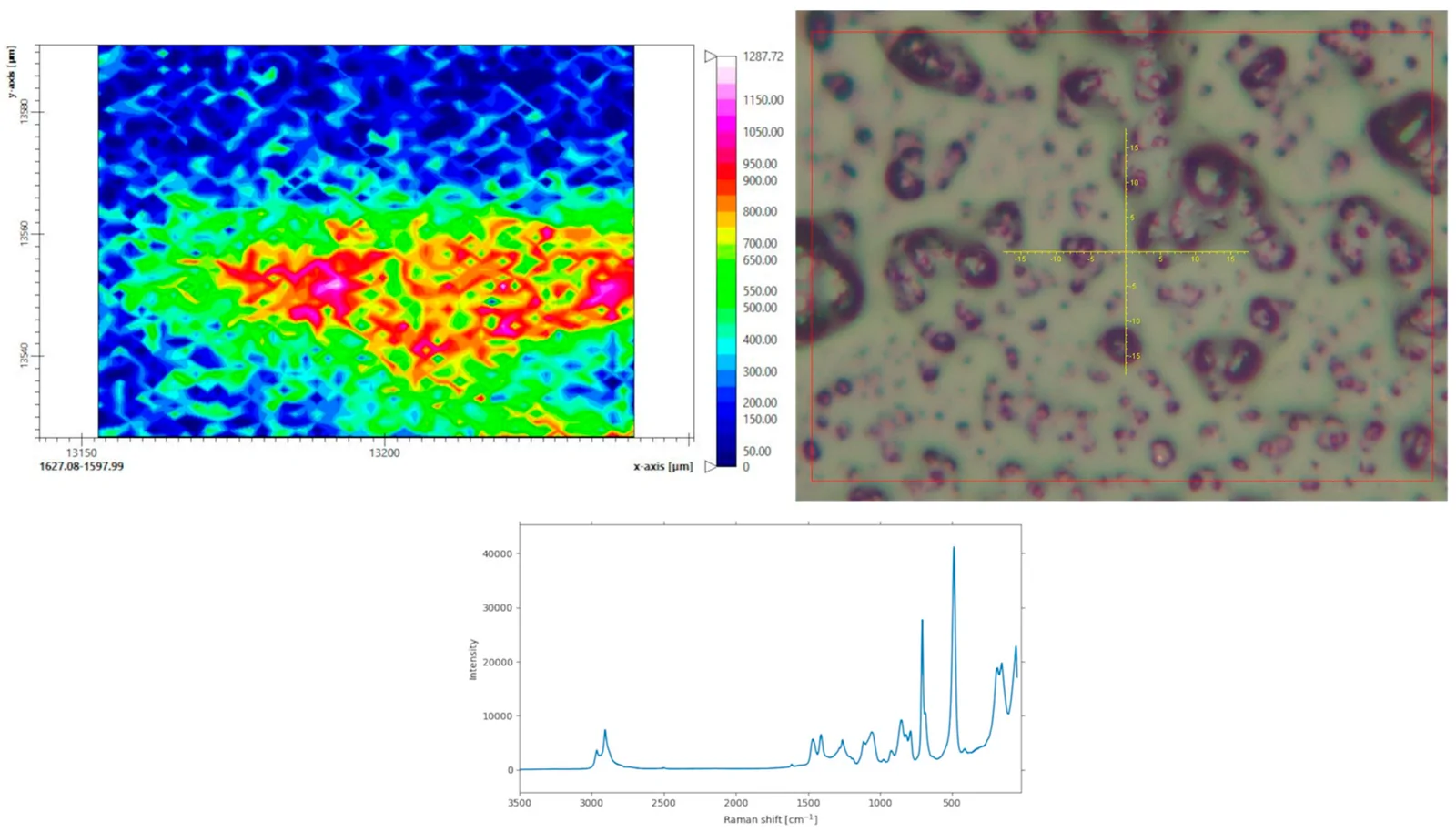
Raman spectrum, chemical map and optical image of the F-9 film.

Raman spectroscopic mapping was used to assess the uniformity of ibuprofen distribution within the glycerol/PEG 200-containing composite films. The fragmented F-6 sample was not mapped because it did not form a continuous film. The Raman maps and corresponding spectra for the continuous F-3 and F-9 films are presented in Figure 6 and Figure 7, respectively. The maps indicate a relatively uniform distribution of ibuprofen within the analysed regions, supporting controlled drug incorporation and helping to explain reproducible apparent release profiles observed for these formulations.

Raman spectroscopic mapping of the functional composite films presented specific experimental challenges. In the F-10 film, which contained sea buckthorn oil, the high concentration of unsaturated lipid components promoted laser-induced photodegradation and local burning under standard mapping conditions (785 nm excitation, >1 mW laser power). Reliable Raman maps could therefore not be obtained for this formulation, even after adjustment of the measurement conditions.

**Figure 8:**
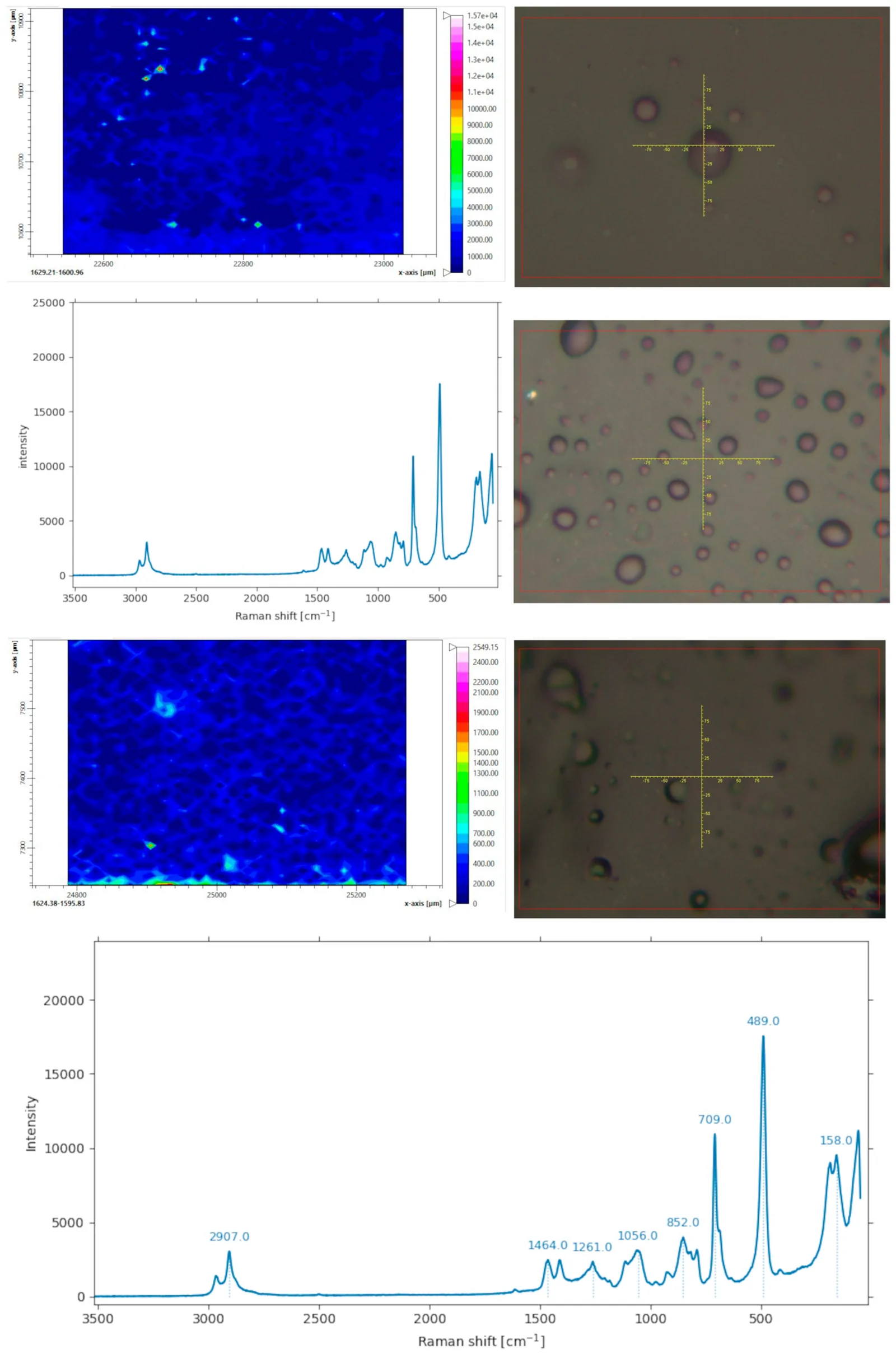
Raman spectra, chemical maps and optical images of the F-11 film recorded at two different points.

Optical microscopy images of the three composite films reveal the presence of droplets of various sizes dispersed within the matrix. These droplets are attributed to hydrophilic polyol-rich phases (glycerol and/or PEG 200) within the hydrophobic silicone polymer network, forming a distinct micro-scale morphology. Crucially, Raman imaging focused on these droplets detected the characteristic ibuprofen peak at 1608 cm^−1^ (associated with aromatic ring vibrations). This finding provides direct visual and spectral evidence that a significant portion of the drug is dissolved and localised within the hydrophilic polyol droplets. These domains may therefore act as microreservoirs for ibuprofen. This phase-separated structure is key to understanding the apparent release mechanism: the drug must first diffuse out of the polyol-rich reservoirs and then through the surrounding silicone matrix to be released, which directly influences the apparent release kinetics and efficiency profiles discussed earlier. The Raman spectra, maps and optical images recorded for the F-11 film are presented in Figure 8.

**Figure 9:**
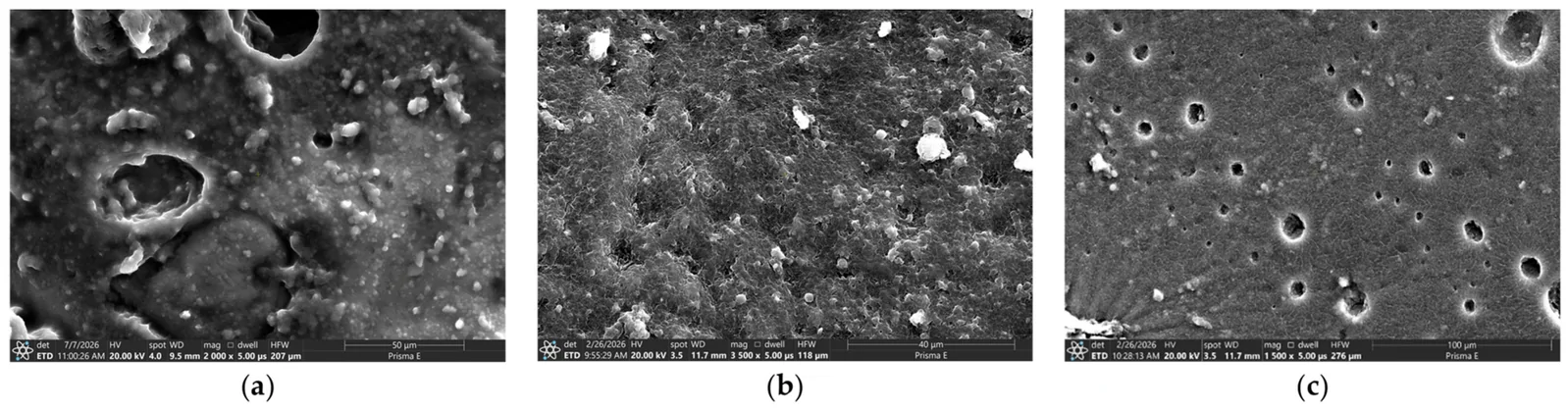
Representative SEM micrographs selected for comparison of the optimised base and functionalised films: (**a**) F-9 control film, (**b**) F-10 film containing CuO nanoparticles and sea buckthorn oil, and (**c**) F-11 film containing Aloe vera extract. Images are shown at representative magnifications; additional SEM images and EDS data are provided in the Supplementary Materials.

For the F-11 film containing Aloe vera extract, as shown in Figure 8 (two different points are presented), the ibuprofen content is relatively uniform within the analysed regions, although part of the ibuprofen may be located below the surface or within deeper polyol-rich domains, unlike the other samples. Representative SEM micrographs comparing the F-9 control and functionalised F-10/F-11 films are presented in Figure 9, with additional SEM/EDS data provided in Figure S2 and Table S4.

Figure 9 compares representative SEM micrographs of the optimised F-9 base film and the two functionalised films. The F-9 control showed a continuous silicone/polyol matrix with dispersed microdomains, consistent with the Raman evidence that hydrophilic domains can act as ibuprofen-rich reservoirs. In F-10, the matrix remained continuous but contained locally distributed particulate and agglomerate-like regions. These features are consistent with the presence of CuO-containing and/or oil-rich domains; the supplementary SEM/EDS evidence and representative elemental composition are provided in Figure S2 and Table S4. The F-11 film displayed more pronounced rounded cavities and surface depressions, which are consistent with the incorporation of an aqueous/polysaccharide-rich Aloe vera phase into the hydrophobic PDMS network. Together with the release profiles, the SEM observations support the interpretation that ibuprofen transport is controlled by a heterogeneous morphology in which domain size, domain connectivity, and matrix hydrophilicity modulate the apparent release rate.

Taken together, the characterisation results provide a coherent but still preliminary mechanistic picture. FTIR indicates ibuprofen-polyol interactions, Raman mapping confirms ibuprofen incorporation and local distribution in selected regions, and SEM shows a heterogeneous morphology with polyol-rich, oil/particle-containing or Aloe vera-derived domains. The apparent release behaviour is therefore most plausibly governed by the combined effects of drug-polyol interactions, domain connectivity, matrix hydrophilicity and diffusion through the surrounding PDMS-rich phase, rather than by a single composition parameter alone.

A limitation of the present materials study is that mechanical and adhesive properties were not quantitatively evaluated. Tensile strength, elongation at break, elasticity, quantitative adhesion, wear-related performance, and long-term stability should be assessed before practical application, as dermal or transdermal films can be considered.

## 3. Materials and Methods

### 3.1. Materials

Ibuprofen was supplied by HuBei Granules-biocause Pharmaceutical Co., Ltd (Jingmen, Hubei, China). The following materials were used as received: hydroxyl-terminated polydimethylsiloxane (PDMSOH, viscosity 2000 cSt, Mw ≈ 36,000 g/mol, Gelest, Morrisville, PA, USA), tetraethoxysilane (TEOS, Sigma-Aldrich, St. Louis, MO, USA), aminopropyl-terminated PDMS (PDMSNH_2_, Mw 850–900 g/mol, Gelest), glycerol (Sigma-Aldrich, St. Louis, MO, USA), 1,2-propylene glycol (Sigma-Aldrich, St. Louis, MO, USA), polyethylene glycol 200 (PEG 200, Sigma-Aldrich, St. Louis, MO, USA), copper(II) oxide nanoparticles (CuO, Sigma-Aldrich), and sea buckthorn oil (LLC Natural Oils, Solnechnogorsk, Russia). Fresh Aloe vera juice was prepared according to a previously reported procedure [35].

### 3.2. Cross-Linking Agents

Silicon glycerate (Si-GL-4) and silicon 1,2-propylene glycolate (Si-PGL-4), without solvated glycol molecules, were synthesised according to previously reported methods [36]. The synthesis and structural confirmation of the combined cross-linking agent, bis(2,3-dihydroxypropoxy)bis(2-hydroxypropoxy)silane (Si-GL:PGL), have been described in our recent publication [37].

### 3.3. In Vitro Apparent Drug Release Studies

#### 3.3.1. Calibration

The calibration curve for ibuprofen in 0.9% NaCl was linear over the concentration range of 0.625–2.1 mg·100 mL^−1^, with R^2^ = 0.9993. The absorbance of the 2.1 mg·100 mL^−1^ standard at 222 nm was 0.81 and was used for concentration calculations in the working range.

The apparent solubility of ibuprofen in the release medium was estimated experimentally. Ibuprofen (1.7 mg) was added to 100 mL of 0.9% NaCl and heated to 37 °C until complete dissolution. The ibuprofen amount was then increased; at 4.3 mg·100 mL^−1^, undissolved crystals were clearly visible. The suspension was filtered and analysed by UV–Vis spectroscopy at 222 nm. The measured absorbance (A222 = 0.46) corresponded to approximately 1.19 mg·100 mL^−1^ (0.0119 mg·mL^−1^), and this value was used as an experimental estimate of ibuprofen solubility under the applied saline-release conditions. Literature data also indicate that ibuprofen solubility is strongly dependent on aqueous-medium composition and pH [20].

#### 3.3.2. Apparent Drug Release

For the in vitro apparent drug release assessment, film samples were placed in tubes containing 5.0 mL of 0.9% NaCl and incubated at 37 ± 1 °C under gentle agitation. At predetermined time points, the release medium was completely withdrawn and replaced with an equal volume of fresh saline. The concentration of released ibuprofen was quantified spectrophotometrically at 222 nm using the established calibration curve. Cumulative apparent release was calculated relative to the total ibuprofen content in each film. All experiments were conducted in triplicate, and the results are reported as mean ± standard deviation. Because of the low experimentally estimated ibuprofen solubility in 0.9% NaCl, the experiment is described as an apparent release study under defined saline-replacement conditions rather than as a strictly sink-controlled release test.

### 3.4. Raman Spectroscopy

Raman measurements were performed using a Bruker SENTERRA II Raman spectrometer (Bruker, Ettlingen, Germany) equipped with an Olympus 50× objective and a 785 nm excitation laser. Spectra were recorded in the range 50–3200 cm^−1^.

For mapping experiments, areas of approximately 90 × 60 μm were scanned with a 2 μm step size and processed using OPUS software (version 8.7, Bruker Optics GmbH, Ettlingen, Germany). The ibuprofen band at approximately 1608 cm^−1^, assigned to aromatic C=C stretching, was selected as the principal marker for chemical mapping because it showed suitable specificity relative to the other formulation components.

### 3.5. FTIR Spectroscopy

FTIR spectra were recorded on an Avatar Nicolet FTIR spectrometer (Thermo Nicolet, Hillsboro, OR, USA) using KBr pellets. Spectra were collected in the range 400–4000 cm^−1^ with a resolution of 4 cm^−1^ and averaged over 32 scans.

### 3.6. Scanning Electron Microscopy (SEM)

Microstructural examinations were performed using a Prisma E scanning electron microscope (Thermo Fisher Scientific, Brno, Czech Republic) equipped with an EDS detector at an accelerating voltage of 10 kV.

### 3.7. Preparation of PDMS Matrices (F-1, F-4, and F-7 Samples)

PDMSOH was mixed with the corresponding cross-linking agent (Si-GL-4, Si-PGL-4, or Si-GL:PGL (2:2)), followed by the addition of ibuprofen (IBU, 0.10 g). After 5 min of mixing, PDMS-NH_2_ was introduced as the RTV catalyst. The mixture was stirred for a further 8–10 min and poured into a plastic Petri dish. The films were allowed to cure at room temperature. The obtained PDMS matrices had a diameter of 4.5 cm and a thickness of 0.75–0.85 mm (Table 1).

### 3.8. Preparation of PDMS/Glycol Composite Films (Samples F-2, F-3, F-5, F-6, F-8, and F-9)

Predetermined amounts of one or more glycols (glycerol and/or PEG 200) were first mixed with PDMS-OH at room temperature. After 5 min of stirring, an emulsion-like mixture was formed. The selected cross-linking agent, PDMS-NH_2_ catalyst, and ibuprofen (IBU, 0.10 g) were then added. The resulting mixture was stirred manually for a further 8–10 min and poured into a plastic Petri dish. The films were allowed to cure at room temperature. The prepared composite films had a diameter of 4.5 cm and a thickness of 1.5–1.9 mm (Table 1).

### 3.9. Incorporation of Functional Additives into F-9 Film (Samples F-10 and F-11)

Two modified composite films were prepared on the basis of the optimised F-9 formulation. Sample F-10 was obtained by introducing a combined dispersion of copper oxide nanoparticles (CuO NPs) and sea buckthorn oil, with a total additive content of approximately 4 wt%, into the standard F-9 mixture prior to curing. Sample F-11 was prepared in a similar manner by incorporating approximately 4 wt% freshly extracted Aloe vera leaf juice into the glycol phase before mixing with PDMS-OH.

For both variants, the cross-linking, catalyst addition, casting, and curing procedures were identical to those used for the base F-9 formulation. The resulting composite films (F-10 and F-11) exhibited a diameter of 4.5 cm and a thickness in the range of 1.74–2.05 mm.

### 3.10. In Vitro Permeation Study Using a Strat-M^®^ Membrane

In vitro permeation testing of the F-9 film was performed using a Franz diffusion cell apparatus (Model 2351-6C) and a Strat-M^®^ synthetic skin diffusion membrane (Millipore^®^, Merck, Merck KGaA, Darmstadt, Germany). The film was fixed over the membrane, and the receptor compartment was filled with 10.0 mL of 0.9% NaCl solution. The temperature was maintained at 37 °C with constant stirring at 800 rpm. At 3, 6, 12, 18, 24, 36 and 48 h, 0.7 mL samples were collected and replaced with an equal volume of fresh 0.9% NaCl to maintain a constant receptor volume and reduce drug accumulation in the receptor phase. The collected samples were analysed spectrophotometrically at 222 nm.

### 3.11. Statistical Analysis

Data are presented as mean ± SD from three independent measurements. Statistical analyses were performed using Python 3.13.5 with NumPy 2.3.5, SciPy 1.17.0, pandas 2.2.3 and statsmodels 0.14.6. Exploratory comparisons of final cumulative release values after 72 h (Q72) were performed within the predefined formulation groups shown in Figure 2 using one-way analysis of variance (ANOVA) followed by Tukey’s post hoc test. Differences were considered statistically significant at *p* < 0.05. In addition, the area under the apparent release curve from 0 to 72 h (AUC0–72) was calculated for each replicate using the trapezoidal rule and analysed in the same predefined groups as a supportive descriptor of the whole release profile. Owing to the exploratory materials-development character of the study and the limited number of replicates, the statistical results were treated as supportive rather than as the sole basis for mechanistic interpretation.

## 4. Conclusions

Ibuprofen-loaded silicone/polyol composite films were successfully prepared using glycerol- and propylene-glycol-derived alkoxysilane cross-linkers. The optimised F-9 formulation combined rapid room-temperature curing with high apparent ibuprofen release (82.91 ± 10.04% after 72 h) and relatively uniform drug distribution. Incorporation of CuO nanoparticles and sea buckthorn oil in F-10 showed the highest numerical cumulative apparent release (94.36 ± 1.79%), whereas Aloe vera incorporation in F-11 produced a pronounced early release stage and a final release of 82.11 ± 14.58%. The release profiles were best interpreted as apparent release under defined saline-replacement conditions because the experimentally estimated ibuprofen solubility in 0.9% NaCl at 37 °C was low. Kinetic analysis supported a dominant diffusion-controlled contribution, with non-ideal/anomalous transport associated with polyol-rich microdomains within the silicone matrix.

The developed films should therefore be regarded as preliminary silicone/polyol materials for local topical/dermal delivery or for further optimisation toward transdermal applications, rather than as validated transdermal therapeutic systems. The low preliminary Strat-M^®^ permeation, absence of biological validation and lack of quantitative mechanical/adhesive testing should be addressed in future studies before practical use can be assessed. The study should therefore be interpreted as a materials-development and preliminary apparent release/permeation investigation, not as biological validation of the final films.

Further development should include biological evaluation, including cytocompatibility, irritation and, where relevant, antimicrobial or wound-healing assays, together with quantitative mechanical and adhesive testing, long-term stability studies and optimisation of skin permeation. These steps will be required to determine whether the platform is best suited for local topical/dermal delivery or can be advanced toward true transdermal administration.

Accordingly, the present study demonstrates a materials-development advance and formulation-screening strategy, while the biological, mechanical and application-related performance of the films remains to be established.

## Figures and Tables

**Table 1:** Composition of ibuprofen-loaded silicone composite films.

Sample	PDMSOH, (g/mmol)	Cross-Linker, (g/mmol)	PDMSNH_2_, (g/mmol)	Hydrophilizing Components, g/mmol	IBU, g/mmol
F-1	1.8/0.05	0.3/0.76 (Si-GL 4)	0.07/0.078	-	0.1/0.49
F-2	1.8/0.05	0.3/0.76 (Si-GL 4)	0.07/0.078	1.8/19.6 Glycerol	0.1/0.49
F-3	1.8/0.05	0.3/0.76 (Si-GL 4)	0.07/0.078	1.35/14.7 Glycerol + 0.45/2.25 PEG 200	0.1/0.49
F-4	1.8/0.05	0.25/0.76 (Si-PGL 4)	0.07/0.078	-	0.1/0.49
F-5	1.8/0.05	0.25/0.76 (Si PGL 4)	0.07/0.078	1.8/19.6 Glycerol	0.1/0.49
F-6	1.8/0.05	0.25/0.76 (Si-PGL 4)	0.07/0.078	1.35/14.7 Glycerol + 0.45/2.25 PEG 200	0.1/0.49
F-7	1.8/0.05	0.275/0.76 (Si-GL:PGL (2:2))	0.07/0.078	-	0.1/0.49
F-8	1.8/0.05	0.275/0.76 (Si-GL:PGL (2:2))	0.07/0.078	1.8/19.6 Glycerol	0.1/0.49
F-9	1.8/0.05	0.275/0.76 (Si-GL:PGL (2:2))	0.07/0.078	1.35/14.7 Glycerol + 0.45/2.25 PEG 200	0.1/0.49

**Table 2:** Main kinetic parameters of apparent ibuprofen release from silicone-based composite films. Q72 values are presented as mean ± SD (*n* = 3).

Sample	Q72 [%]	Zero-Order R^2^	First-Order R^2^	Higuchi kH	Higuchi R^2^	KP * n	KP R^2^
F1	35.54 ± 3.41	0.9749	0.9848	0.0418	0.9922	0.613	0.9984
F2	45.27 ± 6.82	0.9741	0.9744	0.0524	0.9898	0.575	0.9937
F3	70.99 ± 6.00	0.9508	0.9630	0.0824	0.9972	0.528	0.9915
F4	44.34 ± 7.89	0.9667	0.9828	0.0527	0.9939	0.703	0.9717
F5	65.88 ± 7.11	0.9694	0.9787	0.0809	0.9922	0.629	0.9860
F6	77.63 ± 13.54	0.8916	0.9190	0.0883	0.9807	0.527	0.7938
F7	34.46 ± 7.53	0.9790	0.9825	0.0402	0.9888	0.631	0.9966
F8	59.01 ± 11.69	0.9621	0.9756	0.0691	0.9961	0.598	0.9940
F9	82.91 ± 10.04	0.9454	0.9802	0.1004	0.9976	0.673	0.9244
F10	94.36 ± 1.79	0.9470	0.9429	0.1061	0.9952	0.461	0.9922
F11	82.11 ± 14.58	0.8857	0.8976	0.0889	0.9734	0.424	0.8885

* KP = Korsmeyer–Peppas model; KP fitting was performed for the initial apparent release region (F ≤ 0.6). F-6 was not used for direct mechanistic ranking because it did not form a continuous film.

**Table 3:** Main Raman bands of the formulation components relevant to spectral assignment and ibuprofen mapping.

Component	Raman Shift (cm^−1^)	Assignment	Reference
Glycerol	975	CH_2_ rocking	[32]
Glycerol	853	C–O stretching	
Glycerol	789	C–O stretching	
Glycerol	1110	C–C stretching	
Glycerol	1467, 1410	CH_2_ bending	
PEG 200	~845–855	C-O-C stretching, CH_2_ rocking	
PEG 200	~1065, ~1140	C-O/C-C stretching	
PEG 200	~1240, ~1280, ~1345	CH_2_ twisting/wagging	
PEG 200	~1485	CH_2_ bending	
PEG 200	~2875, ~2940	C-H symmetric/asymmetric stretching	
PDMS-OH	~490	Si-O-Si symmetric stretching	[33,34]
PDMS-OH	~710	C-Si-C symmetric stretching	
PDMS-OH	~790	C-Si-C asymmetric stretching/CH_3_ rocking	
PDMS-OH	~1260	CH_3_ symmetric deformation	
PDMS-OH	~1410	CH_3_ asymmetric deformation	
PDMS-OH	~2965, ~2907	CH_3_ stretching (asym. and sym.)	
Ibuprofen (IBU)	~1608	Aromatic C=C stretching	[29,30,31]
Ibuprofen (IBU)	~1455	CH_2_/CH_3_ bending (deformation)	
Ibuprofen (IBU)	~1285	C-O stretching (carboxylic acid)	
Ibuprofen (IBU)	~1180, ~1210	Aromatic C-H in-plane bending	
Ibuprofen (IBU)	~830	Aromatic ring breathing mode	
Ibuprofen (IBU)	~780, ~745	Aromatic C-H out-of-plane bending	

## Data Availability

The original contributions presented in this study are included in the article. Further inquiries can be directed to the corresponding author.

## Supplementary Materials

The following supporting information can be downloaded at: https://www.mdpi.com/article/10.3390/ijms27167446/s1.

## Abbreviations

The following abbreviations are used in this manuscript:

EDS	Energy-dispersive X-ray spectroscopy
FTIR	Fourier-transform infrared spectroscopy
IBU	Ibuprofen
NPs	Nanoparticles
PDMS	Polydimethylsiloxane
PDMS-OH	Hydroxyl-terminated polydimethylsiloxane
PDMS-NH_2_	Amino-terminated polydimethylsiloxane
PEG 200	Polyethylene glycol 200
RTV	Room-temperature-vulcanising
SD	Standard deviation
SEM	Scanning electron microscopy
Si-GL-4	Silicon glycerate cross-linker
Si-PGL-4	Silicon 1,2-propylene glycolate cross-linker
Si-GL:PGL	Mixed glycerol/propylene glycol-derived silicon cross-linker
TEOS	Tetraethoxysilane
UV–Vis	Ultraviolet–visible spectroscopy
