# Supplementary material for: Development of Silicone Elastomer-Based Composite Films Containing Ibuprofen and Functional Additives
Source: Int J Mol Sci. 2026 Aug 20;27(16):7446. doi: 10.3390/ijms27167446 (PMC13513446; doi:10.3390/ijms27167446)
Supplement: Supplementary file 1 [file ijms-27-07446-s001.zip › ijms-4414446-supplementary.pdf]

Supplementary Materials

Development of Silicone Elastomer-Based Composite Films Containing Ibuprofen and Functional Additives

Mari Atabekyan [1], Zoya Farmazyan [1], Nelly Avagyan [1], Vigen Topuzyan [1], Stepan Grigoryan [1], Gohar Khachatryan [2] and Karen Khachatryan [3,*]

[1] The Scientific Technological Centre of Organic and Pharmaceutical Chemistry NAS RA, 26, Azatutyan Str., Yerevan 0014, Armenia; atmari@yandex.ru (M.A.); zoefa2000@yahoo.com (Z.F.); nelli.avagyan80@gmail.com (N.A.); vtop@web.am (V.T.); grigstepan@yahoo.com (S.G.)
[2] Department of Food Analysis and Quality Assessment, Faculty of Food Technology, University of Agriculture in Krakow, Al. Mickiewicza 21, 31-120 Krakow, Poland; gohar.khachatryan@urk.edu.pl
[3] Laboratory of Nanotechnology and Nanomaterials, Faculty of Food Technology, University of Agriculture in Krakow, Al. Mickiewicza 21, 31-120 Krakow, Poland
* Correspondence: karen.khachatryan@urk.edu.pl

The supplementary information contains replicate apparent release data, kinetic fitting details, apparent-release-stage descriptors, and selected additional SEM/EDS evidence supporting the interpretation presented in the main manuscript. No biological validation data are reported in the Supplementary Materials. No mechanical-performance, adhesion-quantification, cytocompatibility, antimicrobial, irritation, or wound-healing data are included; the supplementary data therefore support only the materials-development and preliminary release/permeation interpretation of the study.

S1. Replicate apparent release data

The apparent release profiles were calculated from three independent measurements for each formulation. The values below are reported as mean ± standard deviation (SD). The corresponding profiles in the main manuscript show the mean apparent cumulative ibuprofen release with SD error bars. These values should be interpreted as apparent cumulative release under the defined saline-replacement protocol rather than as intrinsic sink-controlled release performance.

Table S1. Apparent cumulative ibuprofen release values obtained from replicate measurements (mean ± SD, n = 3).

Sample	Release time [h]	Mean apparent release [%]	SD [%]
F1	0	0.00	0.00
F1	0.5	1.68	0.02
F1	2.5	4.03	0.10
F1	4.5	7.15	0.85
F1	24	17.39	1.74
F1	48	26.85	2.71
F1	72	35.54	3.41
F2	0	0.00	0.00
F2	0.5	2.48	0.52
F2	2.5	5.75	0.96
F2	4.5	10.29	1.46
F2	24	21.31	4.69
F2	48	33.86	5.42
F2	72	45.27	6.82
F3	0	0.00	0.00
F3	0.5	4.83	0.76

F3	2.5	11.97	2.62
F3	4.5	19.31	2.49
F3	24	38.13	5.05
F3	48	55.74	4.27
F3	72	70.99	6.00
F4	0	0.00	0.00
F4	0.5	1.14	0.06
F4	2.5	5.38	0.71
F4	4.5	9.83	1.37
F4	24	22.26	4.60
F4	48	34.03	6.85
F4	72	44.34	7.89
F5	0	0.00	0.00
F5	0.5	2.19	0.73
F5	2.5	6.14	1.38
F5	4.5	12.43	3.18
F5	24	30.96	5.17
F5	48	48.55	7.86
F5	72	65.88	7.11
F6	0	0.00	0.00
F6	0.5	6.01	4.95
F6	2.5	20.63	7.17
F6	4.5	30.11	9.14
F6	24	46.03	10.34
F6	48	64.42	9.62
F6	72	77.63	13.54
F7	0	0.00	0.00
F7	0.5	1.38	0.31
F7	2.5	4.04	0.97
F7	4.5	6.92	1.08
F7	24	16.02	3.77
F7	48	25.51	5.91
F7	72	34.46	7.53
F8	0	0.00	0.00
F8	0.5	2.77	1.38
F8	2.5	8.47	2.93
F8	4.5	13.95	4.73
F8	24	30.66	10.10
F8	48	45.18	9.74
F8	72	59.01	11.69
F9	0	0.00	0.00
F9	0.5	3.38	1.33
F9	2.5	13.01	1.18
F9	4.5	20.94	0.51
F9	24	45.75	0.76
F9	48	69.42	10.66
F9	72	82.91	10.04
F10	0	0.00	0.00
F10	0.5	8.73	3.49
F10	2.5	18.70	4.75
F10	4.5	26.61	5.19

F10	24	51.39	2.11
F10	48	71.27	1.44
F10	72	94.36	1.79
F11	0	0.00	0.00
F11	0.5	9.55	2.71
F11	2.5	23.41	5.87
F11	4.5	33.14	7.58
F11	24	48.69	14.28
F11	48	63.86	13.93
F11	72	82.11	14.58

S2. Kinetic model fitting

The apparent cumulative release profiles were fitted using the zero-order, first-order, Higuchi, and Korsmeyer–Peppas equations. Higuchi fitting was used as the principal comparative model for the whole apparent release profile, whereas the Korsmeyer–Peppas model was applied to the initial apparent release region. The Korsmeyer–Peppas fit excluded t = 0 h and used data points up to approximately 60% apparent cumulative release whenever possible. The fitting was used as a comparative and descriptive analysis of the apparent release profiles under identical experimental conditions and was not intended to demonstrate intrinsic release kinetics under strict sink conditions.

Zero-order: $Q(t) = k_0 t + b$

First-order: $Q(t) = Q_{\infty}[1 - exp(-k_1 t)]$

Higuchi: $Q(t) = k_H \cdot t^{1/2} + b$

Korsmeyer–Peppas: $F(t) = k \cdot t^n$; $ln\,F = ln\,k + n \cdot ln\,t$

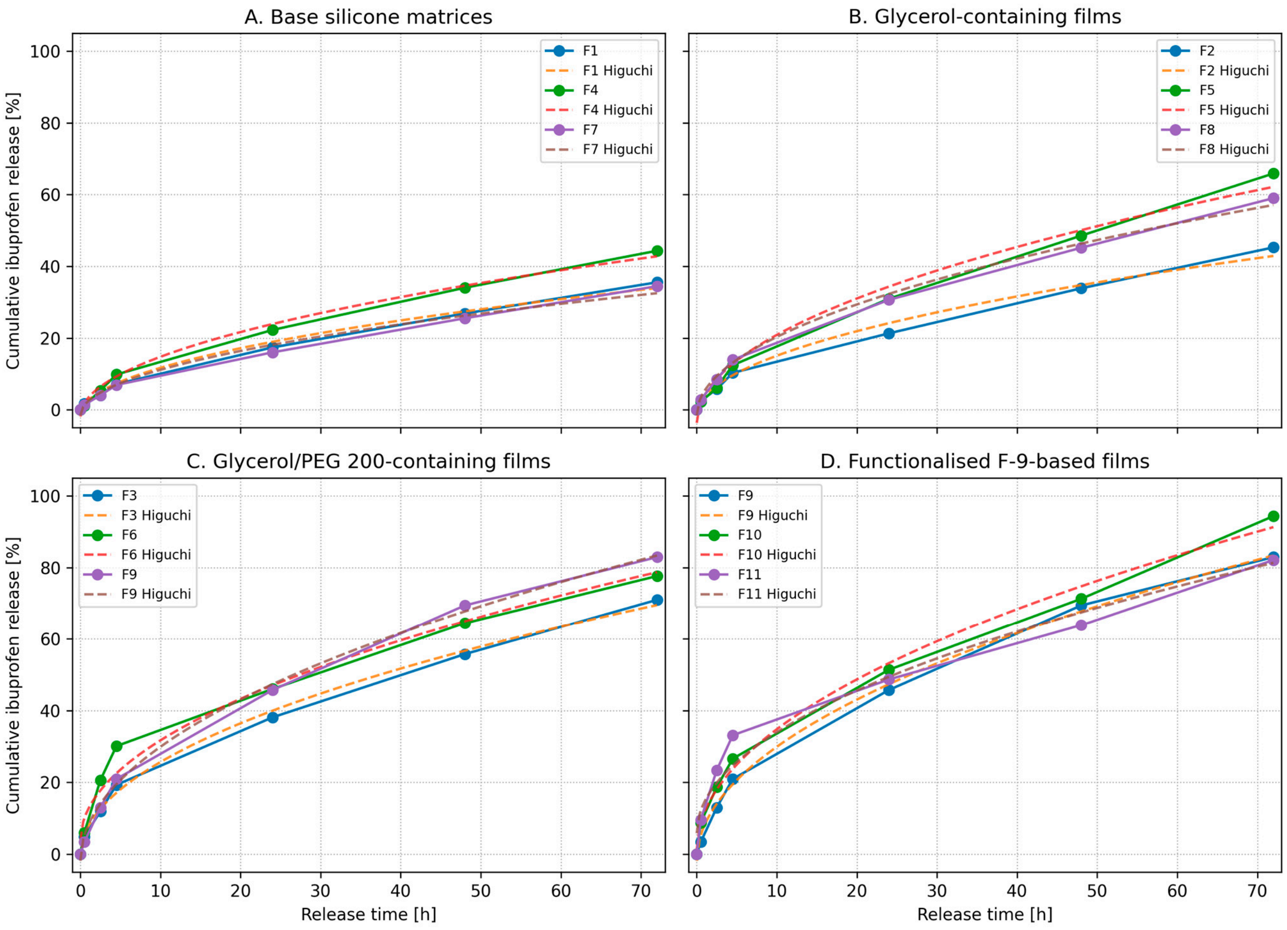

Figure S1. Higuchi fits applied to the mean apparent cumulative ibuprofen release profiles. Solid lines represent mean experimental data, and dashed lines represent Higuchi fits. These fits are provided as supportive descriptors of the apparent release profiles.

Table S2. Full kinetic fitting parameters calculated from mean apparent release profiles. Parameters refer to the mean apparent release profiles.

Sample	Q72 [%]	Zero k0	Zero R^2	First k1	First R^2	Higuchi kH	Higuchi R^2	KP k	KP n	KP R^2	KP domain
F1	35.54	0.4786	0.9751	0.0220	0.9849	4.180	0.9922	0.0254	0.6131	0.9984	$Q \leqslant 60\%$
F2	45.27	0.5993	0.9743	0.0213	0.9744	5.230	0.9897	0.0371	0.5755	0.9936	$Q \leqslant 60\%$
F3	70.99	0.9293	0.9509	0.0343	0.9630	8.239	0.9972	0.0746	0.5282	0.9914	$Q \leqslant 60\%$
F4	44.34	0.5999	0.9666	0.0247	0.9828	5.267	0.9939	0.0244	0.7025	0.9720	$Q \leqslant 60\%$
F5	65.88	0.8923	0.9799	0.0184	0.9879	7.757	0.9879	0.0364	0.6801	0.9928	$Q \leqslant 60\%$
F6	77.63	0.9721	0.8915	0.0908	0.9189	8.827	0.9806	0.1067	0.5268	0.7938	$Q \leqslant 60\%$
F7	34.46	0.4615	0.9791	0.0190	0.9826	4.015	0.9888	0.0228	0.6320	0.9966	$Q \leqslant 60\%$
F8	59.01	0.7845	0.9619	0.0282	0.9755	6.912	0.9961	0.0472	0.5969	0.9940	$Q \leqslant 60\%$

F9	82.91	1.129	0.9455	0.0350	0.9803	10.04	0.9976	0.0627	0.6746	0.9239	Q ⩽ 60%
F10	94.36	1.195	0.9470	0.0375	0.9429	10.61	0.9952	0.1235	0.4609	0.9920	Q ⩽ 60%
F11	82.11	0.9798	0.8858	0.1421	0.8975	8.893	0.9735	0.1458	0.4235	0.8893	Q ⩽ 60%

S3. Two-phase apparent release descriptors

Because only seven sampling points were available, the apparent release profiles were also summarised using simple two-phase descriptors rather than overfitting separate models to each phase. The early phase was defined a–4.5 h and the sustained phase as 4.5–72 h. These descriptors were used only to compare numerical trends in early and later apparent release stages within the same experimental setup.

Table S3. Two-phase apparent release descriptors calculated from the mean release profiles.

Sample	Q4.5 [%]	Q24 [%]	Q72 [%]	Rate 0–4.5 h [% h^{-1}]	Rate 4.5–72 h [% h^{-1}]	Q4.5/Q72 [%]	Q24/Q72 [%]
F1	7.147	17.39	35.54	1.588	0.4206	20.11	48.93
F2	10.29	21.31	45.27	2.287	0.5182	22.73	47.07
F3	19.31	38.13	70.99	4.291	0.7656	27.20	53.72
F4	9.827	22.26	44.34	2.184	0.5113	22.16	50.21
F5	12.43	30.96	65.88	2.761	0.7920	18.86	46.99
F6	30.11	46.03	77.63	6.690	0.7041	38.78	59.29
F7	6.917	16.02	34.46	1.537	0.4081	20.07	46.47
F8	13.95	30.66	59.01	3.099	0.6676	23.63	51.95
F9	20.94	45.75	82.91	4.654	0.9181	25.26	55.18
F10	26.61	51.39	94.36	5.914	1.004	28.21	54.46
F11	33.14	48.69	82.11	7.365	0.7254	40.37	59.30

S3.1. Exploratory statistical analysis of Q72 and AUC0–72

Exploratory statistical comparisons were performed only for the final apparent cumulative release values after 72 h (Q72) and for the area under the apparent release curve from 0 to 72 h (AUC0–72). The comparisons were restricted to the predefined formulation groups used in Figure 2 of the main manuscript. One-way ANOVA followed by Tukey's post hoc test showed no statistically significant differences within these groups at $p < 0.05$. Therefore, the differences between formulations are discussed in the manuscript as numerical trends obtained under the same saline-replacement protocol, not as statistically confirmed differences in intrinsic release performance.

Table S3a. Exploratory statistical comparison of Q72 and AUC0–72 within the predefined formulation groups.

Group	Formulations	Q72 ANOVA p	AUC0–72 ANOVA p
A	F1 / F4 / F7	0.213	0.176
B	F2 / F5 / F8	0.071	0.147
C	F3 / F6 / F9	0.421	0.197
D	F9 / F10 / F11	0.331	0.706

The statistical treatment was therefore used as supportive evidence only. No significance letters were added to the apparent release plots because no significant pairwise differences were detected in the predefined groups.

S4. Experimental estimate of ibuprofen solubility in saline

Ibuprofen solubility in the release medium was experimentally estimated in 0.9% NaCl at 37 °C. Initially, 1.7 mg of ibuprofen was added to 100 mL of 0.9% NaCl and dissolved at 37 °C. The ibuprofen amount was then increased; at 4.3 mg/100 mL, undissolved crystals were clearly visible. The suspension was filtered and analysed by UV–Vis spectroscopy at 222 nm. The measured absorbance was A222 = 0.46. Using the calibration response described in the main manuscript, this corresponded to approximately 1.19 mg/100 mL, equivalent to 0.0119 mg/mL. This value was used as an experimental estimate of ibuprofen solubility under the applied release-test conditions. This low apparent solubility supports the interpretation that strict sink conditions were not achieved, even though complete medium replacement reduced drug accumulation during the experiment.

S5. Supplementary SEM and EDS evidence

Additional SEM micrographs were selected to support the morphological interpretation in the main text without overloading the main manuscript with all recorded images. The selected images show the F-9 control morphology, the particulate/domain-rich surface of F-10, the microcavity-rich morphology of F-11, and EDS evidence for copper-containing domains in the CuO-containing F-10 formulation. These images are used only to support morphology-based interpretation of apparent release behaviour and do not provide evidence of cytocompatibility, antimicrobial activity, irritation potential, or wound-healing performance.

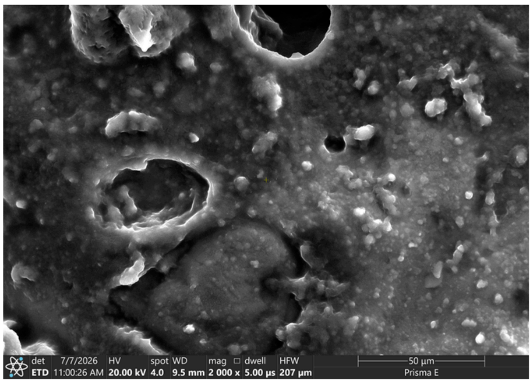

(a) F9 control, medium magnification

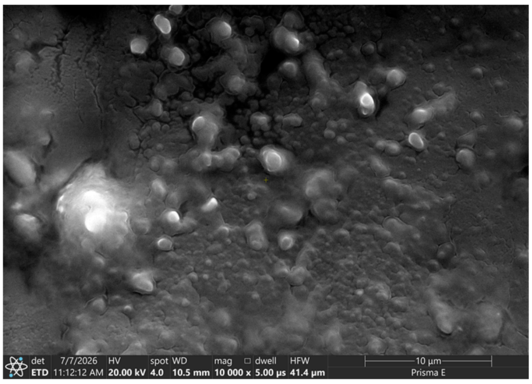

(b) F9 control, higher magnification

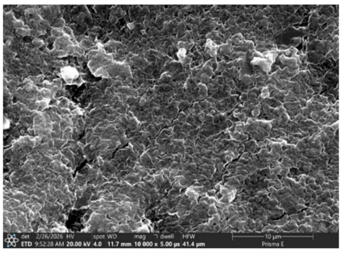

(c) F10, surface morphology

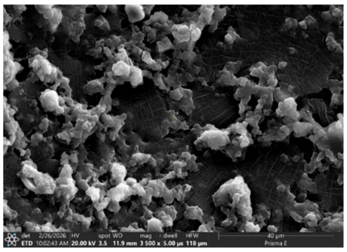

(d) F10, particulate domains

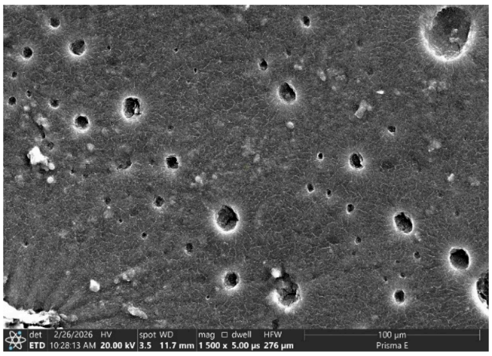

(e) F11, microcavities

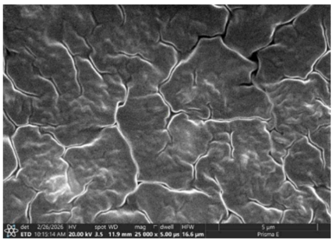

(f) F11, surface fissures/domains

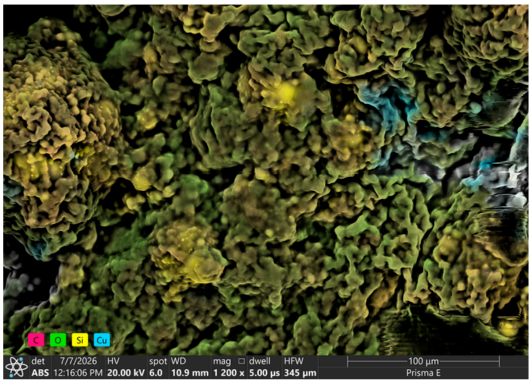

(g) EDS/Cu-containing domains

Figure S2. Selected supplementary SEM/EDS images: (a,b) F-9 control film; (c,d) F-10 film containing CuO nanoparticles and sea buckthorn oil; (e,f) F-11 film containing Aloe vera extract; and (g) representative EDS/Cu-containing domain map.

S6. EDS summary for copper-containing regions

The EDS results confirmed low but detectable copper levels in selected analysed regions. Because EDS is local and semi-quantitative, the values should be interpreted as supporting evidence for Cu-containing domains rather than as bulk composition. Detection of copper-containing regions confirms the presence of the inorganic additive locally, but should not be interpreted as evidence of antimicrobial or biological activity of the film.

Table S4. Representative EDS elemental composition of analysed Cu-containing regions.

Region	C [at%]	O [at%]	Si [at%]	Cu [at%]	Cu [wt%]
1	47.1	50.7	2.1	0.1	0.4
2	50.6	45.6	3.6	0.1	0.6
3	46.4	50.9	2.7	0.1	0.2
4	48.8	47.7	3.3	0.1	0.4
5	47.4	49.7	2.7	0.1	0.5
Map/area	50.7	43.8	5.2	0.2	1.0
